# The Experiences and Challenges Encountered by Tow Truck Driver's Attending Roadside Events: A PRISMA Scoping Review

**DOI:** 10.1002/puh2.70296

**Published:** 2026-06-12

**Authors:** Brenda Ruvimbo Mutsvairo, Daniel Terry, Blake Peck, Liz Ryan

**Affiliations:** ^1^ School of Nursing and Midwifery University of Southern Queensland Toowoomba Queensland Australia; ^2^ Institute of Health and Wellbeing Federation University Ballarat Victoria Australia; ^3^ School of Nursing and Midwifery Deakin University Melbourne Victoria Australia

**Keywords:** mental health, physical, psychological, roadside events or crash events, safety, tow truck drivers, trucking industry, well‐being

## Abstract

**Introduction:**

Tow truck drivers (TTD) are often the first to present at vehicle crashes, in addition to police, ambulance and at times fire services. Existing research suggests that TTD do not always receive similar supports as other first responders. A gap highlighted the need to understand the physical and psychological experiences of TTD, how they manage challenges and what supports they receive. The aim of the study is to understand why TTD should be considered first responders and to explore experiences related to the challenges encountered in their work.

**Methods:**

A scoping review was conducted to address the aims of the study using the PRISMA for scoping reviews guidelines to ensure accurate and rigorous reporting of findings. A broad literature search was conducted between February and April 2024, using the databases PubMed, Scopus, CINAHL and EBSCO (Newspaper Source Plus), while also searching Google Scholar search engine to identify sources related to TTD. Studies of TTD were included if they were original research, written in English, peer‐reviewed or news reports. Studies were excluded if truck drivers were not related to the tow truck industry.

**Results:**

A total of 14 studies were included in the review. No specific key attributes for TTD were reported; however, they were found to be the first on the scene following an accident due to their well‐developed network. TTD experience physical and psychological trauma. Support services are limited, and poor coping strategies, such as excessive alcohol intake, are reported. Lack of safety training was considered a contributor of occupational hazards in the industry.

**Conclusions:**

There is a palpable inequity of supports available to this group of first responders when compared to other common disciplines. The findings support a deeper exploration of TTD's lived experiences on the job, including how best to address their physical and psychological well‐being.

## Graphical abstract

The review demonstrates the challenges that are experienced by Tow Truck Drivers (TTD) during their daily roles which includes attending crash scenes. One of the challenges is limited safety training which affects their performance and puts them at risk of being injured physically and psychologically. However, formal first responders attend to similar crash scenes as TTD and receive different support. Therefore, the outcome is to improve support services for TTD.

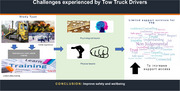

## 1 Introduction

Tow truck drivers (TTD) attend almost all car crashes and provide various roadside services to motorists, such as towing and repairing vehicles and moving damaged vehicles from roadsides to depots and repair locations [1, 2]. Much of what TTD do is also in collaboration with first responders, such as police or paramedics, when attending motor vehicle crashes [2].

Historically, first aid was not performed by TTD due to potential legal consequences in the case of the injury worsening [1]. However, Dean et al. [1] recommended first aid practice to assist individuals in critical motor vehicle collisions. TTD are now trained to perform first aid and cardiopulmonary resuscitation (CPR) when individuals require medical attention [3].

TTD face significant daily risks, including occupational injury and death, resulting from being struck by passing vehicles while working on busy and hazardous roadways [2]. In addition, they also encounter traumatic events, such as seeing dead or injured bodies and extricating injured individuals from vehicles to prevent death [4].

Although there is some evidence that companies provide support to their employees, there remains a lack of understanding about how TTD manage the challenges of their work [5]. Personal responsibility for well‐being, access to appropriate mental health pathways (such as referral systems, support services and treatment options) and inclusive workplace practices are encouraged as key strategies to support the mental health of TTD [5]. Currently, the tow truck industry has the highest fatality rate both physically and psychologically in the context of witnessing fatal car crashes or witnessing colleagues being injured or killed during work when compared to fire and police service [6]. There is a mismatch of available supports despite also attending road accidents and experiencing physical, verbal and emotional trauma through the work they undertake [2, 7].

Although TTD frequently arrive at crash sites alongside police, ambulance and at times fire services, they remain significantly understudied in the literature. Evidence suggests a rising number of road crashes globally, along with a high dependency on towing services to manage emergency situations and restore traffic flow. Yet, TTD continue to lack formal acknowledgement within occupational health and safety policies despite their crucial frontline role. Recognising TTD as adjacent or auxiliary first responders is essential, not only to validate their frontline role but also to ensure they receive appropriate support, resources, and protections. This recognition is a necessary step toward improving their mental health outcomes, workplace safety, and integration into broader emergency response systems. There remains very little research examining their role and ongoing needs, including how they should be situated among first responders. Therefore, a scoping review was conducted to map the existing literature, identify key concepts and highlight gaps.

### 1.1 Aim

This scoping review aims to explore the occupational experiences and challenges faced by TTD in the context of motor vehicle crashes, focussing on physical and mental health, well‐being and access to support services. A secondary aim examines the extent to which TTD are recognised as first responders, and whether their role should be formally acknowledged. This review seeks to identify gaps in knowledge and inform future research to improve recognition and support for this essential workforce.

## 2 Methods

The scoping review followed both the PRISMA for scoping reviews (PRISMA‐ScR) guidelines and the Arksey and O'Malley [8] framework to ensure methodological rigour and academic transparency. PRISMA‐ScR provided a structured approach for planning, conducting and reporting the review, whereas Arksey and O'Malley guided the iterative process of defining research questions, identifying relevant studies and applying screening criteria [8]. This combined framework ensured clarity in inclusion and exclusion decisions and supported a comprehensive, reproducible synthesis of evidence. Narrative synthesis was used to evaluate and synthesise findings from multiple studies [9]. The PRISMA‐ScR guidelines were followed to ensure accurate and complete reporting of findings [10]. Additionally, the PRISMA‐ScR was conducted to identify and analyse the current knowledge related to TTD [11].

### 2.1 Search Strategy

A literature search was conducted between 19 February and 30 April 2024, with an additional search being conducted on 30 September 2025, using PubMed (*n* = 430), Scopus (*n* = 267) and CINAHL (*n* = 109), EBSCO (Newspaper Source Plus) (*n* = 3216) and Google Scholar (*n* = 100), due to its coverage of academic information which covers most fields [12]. An academic librarian was consulted to ensure key database selection and accuracy, enabling optimal outcomes. Results were assessed using title and abstract, followed by full text (Supporting Information file 1).

### 2.2 Inclusion and Exclusion Criteria

Studies were included if they were original research, written in English, peer‐reviewed or news reports. Towing company and legal website reports were also included, if they were centred on day‐to‐day operations of TTD studies that encompassed roadside services, safety and first response were included. The search covered literature published between 1975 and 2025 to ensure a comprehensive review of the past 50 years. Dean et al. [1] provide seminal accounts of the roles and responsibilities of TTD in motor vehicle crashes. Studies were excluded if truck drivers were not related to the tow truck industry. Further, grey literature was excluded due to insufficient data regarding the experiences of TTD. Articles not in English were excluded due to difficulties with translation.

### 2.3 Study Screening

EndNote (version 21) was used to manage records and remove duplicates. Subsequently, one reviewer (BM) conducted an initial relevance check to remove articles that were clearly outside the scope of the review. Two reviewers (BPand DT) then independently screened all remaining titles, keywords and abstracts against the predefined inclusion and exclusion criteria as noted earlier, focusing on population, context, study design and relevance to the review question.

Full‑text articles that appeared eligible were retrieved and assessed in detail by two reviewers (LR and DT), who again applied the same criteria to confirm eligibility, including verification of methodological relevance and alignment with the review's conceptual boundaries. Any disagreements at either stage were discussed and resolved with a fourth reviewer (BP) until consensus was reached.

### 2.4 Methodological Quality Assessment

In accordance with established scoping review methodology, a formal assessment of methodological quality or risk of bias was not undertaken. ScR are designed to map the breadth and nature of available evidence rather than to evaluate the quality of individual studies and appropriate when the literature is limited or heterogeneous, as is the case in this review [10, 13].

### 2.5 Data Extraction and Analysis

Given the diversity of the data, textual data extraction into Microsoft Word data analysis was undertaken to facilitate Braun nd Clarke's [14] method of thematic analysis. Findings were grouped into other similar topics and domains, leading to the identification of overarching themes. This exercise was to propose meanings of the data and to be empathetic of others’ experiences [15] (Supporting Information file 2).

## 3 Results

**FIGURE 1:**
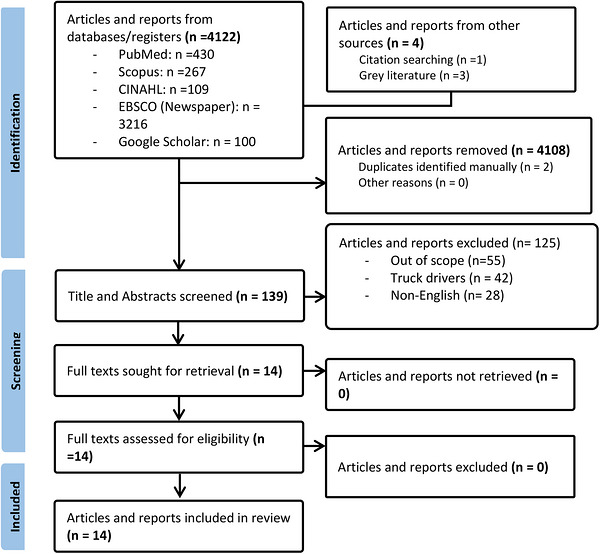
PRISMA‐ScR Flow Chart.

The literature search yielded 139 potentially relevant publications. A total of 14 studies were agreed upon for inclusion in the review (Figure 1). Overall, the final group of publications included four (*n* = 4) case control studies [1, 2, 16, 17] and eight (*n* = 8) narrative reports [4, 5, 7, 18, 19, 20, 21, 22, 23, 24]. Three were Australian [1, 4, 5], one from Canada [22], and the remainder from the United States [2, 7, 16, 17, 18, 19, 20, 21, 23, 24] (Table 1).

**TABLE 1:** Key literature of the review.

References	Origin and type of literature	Methodology and data collection and analysis	Sample size and population	Findings
Bohn [23]	USA Legal website	Narrative. Data are based on a law firm perspective	No sample	TTD may cause accidents due to fatigue, increased speed, alcohol and drug use, and inadequate safety checks of the tow truck Effects of tow truck crashes include fractures, spinal cord injuries and traumatic brain injuries Advise to seek medical attention even without injuries Seeking legal advice post‐accident
Bunn et al. [16]	USA Peer‐reviewed	Case control studies. Data collected from the Kentucky FACE programme. Injury scenario text analysis	No sample size but sources of data included media reports, collision report analysis for safer highways (CRASH) and death certificate data for 2005 to 2016	29 traffic incident management (TIM) fatalities 20% of the TIM fatalities occurred in the fire protection and motor vehicle towing industries The highest percentage of pedestrian death was in towing and recovery occupations (83%) Motor vehicle towing industry was identified as high risk for occupational fatalities
Calligeros [4]	Australia news report	Narrative. Data are from a news report	No sample	TTD witness traumatic events but get no support Risk of assisting individuals known to the TTD Lack of government support to manage the mental well‐being of TTD Impacts of the traumatic events include substance misuse, alcohol abuse, PTSD and depression Culture of stigma and expectation to be ‘tough’ in the industry limit help‐seeking
CDC [18]	USA Government report	Narrative report. Data were collected from a witness at the scene of the accident	No sample	Death of the towing company owner whilst connecting the car to the tow truck No official safety training in the company Incident occurred in the snow where the truck slid Wife witnessed the crash of her husband (TTD)
CDC [19]	USA Government report	Narrative report. Data included a report from the owner of the towing company and the box truck driver	No sample	A 22‐year‐old male TTD was struck by a passing box truck leading to death Contributing factors included the tow truck being close to passing traffic and a narrow highway shoulder Safety programs and training were conducted daily and within 4 weeks of commencement on‐the‐job, respectively Weather: clear skies and no wind but there was snow 3 weeks leading up to incident
Chance's Truck and Auto Salvage [20]	USA Tow truck company report	Narrative. Data obtained from a Tow Truck company	No sample	TTD experience stress when driving in harsh weather conditions Lack of sleep and fatigue increase the risk of personal safety due to long hours of work (burnout) Safety is compromised when other motorists drive poorly
Chandler and Bunn [2]	USA Peer‐reviewed	Case control study. Data were collected from the Occupational Safety and Health Administration (OSHA) IMIS database. Coded and narrative analysis	106 cases. Information collected included demographics, injury details, establishment details, violation details and investigation details	92% of the cases were male 105 cases were established as private owned and 1 local government owned Caught‐in and struck‐by injuries were the most causes of fatal and non‐fatal injuries 79% of the victims were struck on a 2‐way divided roadway and 21% were struck on a 2‐way undivided roadway Fall season had the most pedestrian struck‐by injuries compared to spring which had the least
Dean et al. [1]	Australia Peer‐reviewed	Case control study. Data were collected by interviews, a month's records of a company and observations Analysis is unclear	19 firms. Managers and drivers, and a towing firm. Records of a company were included	Trucks were distributed to strategic place with a high occurrence of a crash Annual inspection of winch and towing equipment The TTD is usually the first on the scene and calls the ambulance Lack of emergency vehicle status of the tow truck TTD feared use of their first aid training due to liability concerns
Madden [5]	Australia Tow truck company report	Narrative report. Data are based on day‐to‐day experiences of a towing company	No sample	TTD witness distressing events, such as injuries or death Harvey's Towing promotes mental well‐being Tries to reduce stigma and provides referrals for support, such as GP and websites Provides debrief and attends workshops to learn more about mental health
Pitt [7]	USA Legal website	Narrative. Data obtained from a law firm	No sample	TTD experience mental and financial constraints Risk for physical comorbidities, such as muscle sprains, due to heavy lifting to hook up vehicles Risk of violence from the individuals TTD assist
Resch [21]	USA Tow industry website	Narrative report. Data are based on an individual's experience	No sample	Tow trucks are not considered emergency vehicles Assumptions that TTD are emergency responders due to driving behaviours Lack of formal high‐speed training to attend emergency tow truck response Important to know what the state legislation deems acceptable as emergency vehicle for TTD
Sanderson [22]	Canada News report	Narrative report. Data collected from individual's experiences	No sample	Response to wrecked cars risks psychological distress and this is overlooked by the government Lack of training to manage traumatic incidents Difficulty with forgetting crash events even after years of occurrence Need for employee programs to offer training
Tefft et al. [24]	USA Traffic safety report	Case control study. Data were collected from the National Tow List and Emergency Responder Safety Institute (ERSI). Descriptive analysis	123 roadside assistance providers (RAP). Information included circumstances of death for tow operators and a list of struck‐by‐vehicle fatalities of all types of emergency responders for years 2015–2021	123 deaths due to being struck by vehicles Weather and roadway surface conditions contributed to where the RAPs were struck RAPs were struck whilst working in the travel lanes, collision of RAP vehicle resulting in RAP being struck and killed Drivers who fatally struck RAPs left the scene of the crash Drivers who struck the RAPs included some who were fully licensed and intoxicated with alcohol
Yang et al. [17]	USA Peer‐reviewed	Case control study. Data were collected from news reports managed by the ERSI. Text mining analysis	5113 responder‐involved incidents between 11 July 2001 and 6 December 2020. Information was obtained for line‐of‐duty deaths (LODD), near miss and stuck‐by incident news	Law enforcement agencies comprised of the largest proportion of LODD and struck‐by incidents TTD comprised of 20% LODD and approximately only 3% of struck‐by incidents Most incidents occurred in a travel lane followed by the shoulder, over 13% and 5%, respectively Limited attributes for weather conditions but the most frequent was snow followed by rain Extreme emergency lighting caused distraction leading to crashing with responders

Abbreviations: PTSD, post‐traumatic stress disorder; TTD, tow truck drivers.

**FIGURE 2:**
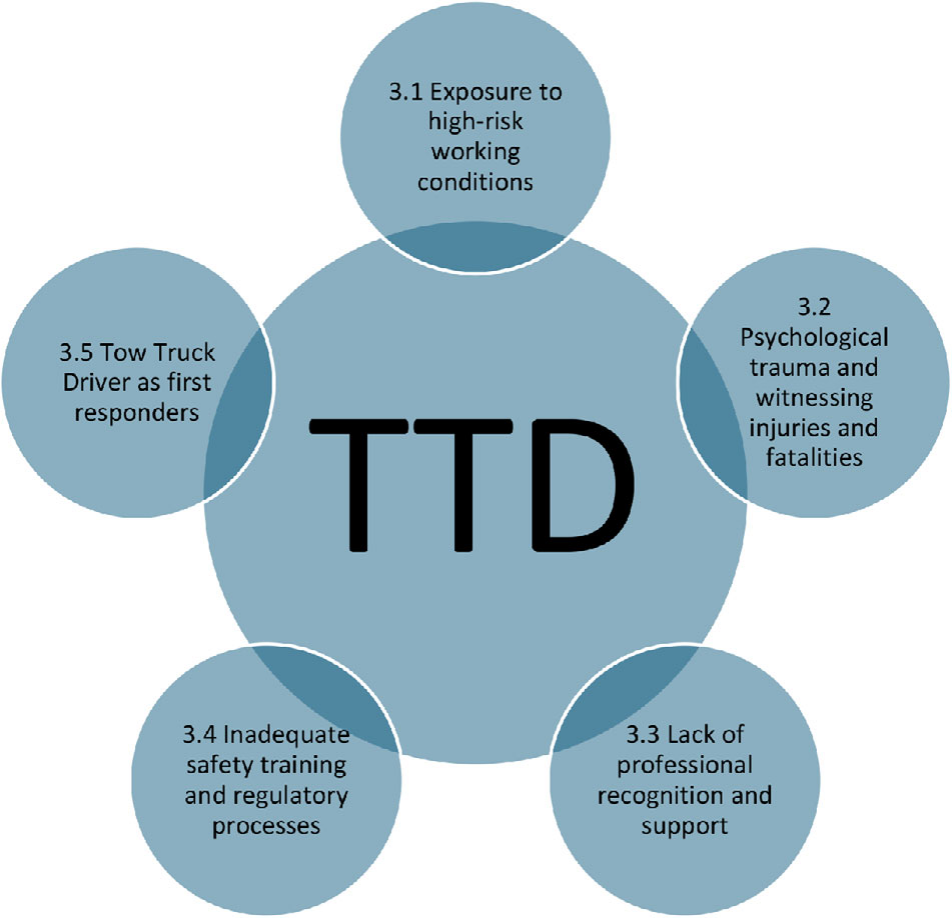
Visualisation of identified themes. TTD, tow truck drivers.

Among the 14 identified articles and reports, five themes were identified for the lived experience of TTD. These themes encompass physical trauma, psychological trauma, coping and support services, safety training and being defined as first responders (Figure 2) (Supporting Information file 3). Each of these five themes is discussed in detail.

### 3.1 Exposure to Trauma and High‐Risk Working Conditions

Collective studies in the United States indicate that during the day‐to‐day operation of tow truck driving, TTD can experience physical trauma by being struck by other vehicles, crushed beneath vehicles, assault from consumers and tow truck accidents [2, 7, 16, 18, 23].

According to Tefft et al. [24], there were 123 roadside assistance worker fatalities in the United States between 2015 and 2021, equivalent to one every 17.8 days. However, these figures may underestimate the true toll due to challenges with data accuracy and underreporting [24]. More recent data from the Emergency Responder Safety Institute (ERSI) suggest even higher fatality rates, with 155 emergency responders killed between 2019 and 2021, and an additional 27 deaths reported by the end of June 2022 [17]. All incidents were classified as ‘struck‐by’ deaths, near‐miss fatalities or line‐of‐duty deaths, indicating a TTD death approximately every 9.4 days [17].

Chandler and Bunn [2] in the United States reported 231 work‐related deaths in the motor vehicle towing industry between 2011 and 2017. Overall, there is ongoing global underreporting of work‐related injuries and diseases, however, further obscuring the true scale of risk faced by TTD [16]. Therefore, understanding the underlying causes of these incidents is essential to developing effective strategies that reduce their occurrence and improve safety outcomes.

The majority of the studies regardless of country reported contributing factors such as being struck while servicing the vehicle, struck by equipment or a moving vehicle, such as when the TTD is under the vehicle connecting it to the tow truck, failure to set the parking brake or the unexpected rolling of the vehicle [2, 18]. Additionally, environmental factors, such as ice, may cause vehicle movement leading to death [18]. Another factor contributing to injury is the jacking equipment which lifts and supports the car and when the vehicle moves from its block support, potentially causing fatality [2].

In Australia, TTD's injury or death occurred due to failure of equipment or being crushed between equipment [25, 26]. A repeated finding across multiple studies includes fatalities that occurred when TTD were struck by moving vehicles working on the side road, exiting or entering the car, providing directions and working close to passing traffic [2, 19, 24]. Bunn et al. [16] found 17 deaths occurred to individuals conducting work outside of the vehicle, and over 50% were struck by other vehicles. Moreover, Tefft et al. [24] reported a third of the struck victims were standing or operating in the travel lanes, and 63% were working outside the shoulder road. Poor driving from other motorists who do not stay in their lane can also provoke such fatalities [20].

Connecting vehicles and lifting heavy machinery predisposes TTD to musculoskeletal injuries, fractures, traumatic brain injury and spinal cord injuries [7, 23]. Chandler and Bunn [2] reported TTD being caught between or under moving mechanical equipment, resulting in injury, including lacerations, fractures, amputations and asphyxia. Several studies reported that TTD also face physical trauma from assaults by angry motorists, burnout‐induced collisions from long work hours and crashes caused by impaired, distracted, speeding drivers or brake failures [7, 17, 23].

### 3.2 Psychological Trauma and Witnessing Injuries and Fatalities

Psychological trauma is also experienced among TTD, where they may witness distressing accidents leading to mental trauma [5]. Calligeros [4] and Sanderson [22] in Australia and Canada, respectively, found witnessing accidents leave TTD shaken, stressed and disturbed in the short and long term, potentially suffering post‐traumatic stress disorder (PTSD). Factors contributing to psychological trauma include TTD witnessing fatal crashes or observing, retrieving or cleaning up human remains from within vehicles and on roads [4].

TTD reported the preconceived trauma of having to rescue family or friends and an actual rescue of a friend's son. TTD described witnessing more death and dead bodies than the average military personnel during active duty [4]. Diverse literature outlined that family members or co‐workers of TTD may also experience psychological trauma through witnessing their TTD colleagues or family members being physically injured [16, 18]. TTD have also been called as witnesses for coroner's reports, and narrating traumatic incidents may cause further psychological distress [16]. Multiple authors regardless of country reported TTD to have higher fatality rates from physical and psychological injuries compared to fire and police professions [4, 16, 22].

### 3.3 Lack of Professional Recognition and Support

Beyond the physical and psychological impacts of working in the tow truck industry, an additional theme identified the coping mechanisms and support services available to them. Despite having one of the highest fatality rates, it was commonly reported among different countries that the tow truck industry remains largely under‐recognised by governments in regard to funding [4, 16, 22]. Sanderson [22] reported that TTD have ‘thick skin’ (Para. 3) and ‘immunity’ (Para. 14), therefore perceived to have a high level of resilience to what they witness and experience when dealing with road traumas [4].

A recurring finding between Calligeros [4] and Pitt [7] includes a lack of formal emotional and mental health support systems, and mandatory counselling remains problematic. Without these supports, TTD may develop PTSD, helplessness, depression, insomnia and a lack of coping strategies or ability to address the trauma [4, 7]. Reports described TTD as the ‘forgotten victims’ of road traffic collisions due to their lack of support, barriers to seeking help and maladaptive coping strategies, including drug and alcohol misuse, which may occur during working hours [4, 17].

Barriers to seeking help, such as stigma and workplace culture, exacerbate psychological stress [4], and enablers, such as inclusiveness, well‐being, reducing stigma and mental health promotion, remain minimal [5]. In some areas of Australia, education has been provided to enhance mental health knowledge among TTD, but these practices remain inadequate and sporadic, with a localised approach [5]. Services and resources, such as websites, or referrals to family physician or general practitioner, medical specialists and online support, may be provided by a specific tow truck company, but standard national approaches are largely absent [5].

### 3.4 Inadequate Safety Training and Regulatory Protections

Safety training also emerged as a key theme concerning TTD. As outlined previously, physical and psychological injuries can result from the equipment used during vehicle recovery [2, 24]. Chandler and Bunn [2] identified the lack of comprehensive safety training as a contributing factor to occupational hazards within the industry. Mental health safety also remains under‐addressed, making it difficult for drivers to cope with the emotional toll of attending distressing crash scenes [22].

Training programmes can benefit towing personnel by improving preparedness and reducing risk of injury. Therefore, routine health and safety checks, including regular preventative maintenance, are conducted on tow trucks in the United States [19]. United States and Australian literature suggested that compliance with roadworthiness standards and possession of appropriate licensing be mandatory [1, 19].

TTD encounter significant safety risks when attending crash scenes. Bunn et al. [16] and Chandler and Bunn [2] state the use of orange flashing lights on tow trucks is an insufficient alert for motorists to slow down or give way, especially in high‐speed environments, and can be obstructed during vehicle loading, further compromising visibility. Moreover, in Australia and the United States, TTD typically do not receive high‐speed driving training yet are expected to respond promptly to incidents, potentially delaying arrival and elevating risk [1, 21].

Several environmental and situational factors within the United States pose safety threats, such as time of day, weather conditions and road infrastructure. Chandler and Bunn [2] reported 43% of TTD incidents occurred in darkness, a finding echoed by Yang et al. [17]. Consolidated findings include adverse weather—such as snow, rain, fog and storms—further compromises safety [17, 18, 19, 24]. Icy roads can cause vehicles to shift unexpectedly, causing injuries [18]. Collective literature reported road conditions, such as narrow shoulders, potholes and debris, also present ongoing hazards [19, 20].

### 3.5 TTD as First Responders

Although no formal definition or set of key attributes for TTD as first responders was identified, understanding their role is essential to evaluating their potential inclusion in this definition. Dean et al. [1] indicated that TTD often arrive at crash scenes before police or paramedics, due to their well‐established dispatch networks. This was echoed by Yang et al. [17], who classified TTD as roadside emergency responders, suggesting a broader recognition of their role in incident response.

In crash scenarios, TTD frequently work alongside police, paramedics and firefighters, reinforcing their functional proximity to traditional first responders. Literature findings indicate a growing notion that TTD should be considered first responders, given their rapid arrival at crash scenes and their involvement in managing high‐risk environments [1, 21]. However, towing services are not currently recognised as authorised emergency vehicles. Calls have been made for policy changes, such as the introduction of special sirens, to improve their visibility and safety during emergency response [21].

Within the broader framework of transportation incident management (TIM) in the United States, TTD are grouped with police, firefighters and emergency medical personnel, professions already acknowledged as first responders [16]. Yet this classification varies across countries. In Australia, Dean et al. [1] proposed that TTD form part of a ‘quartet’ of first responders due to their rapid response capabilities.

Despite these arguments, literature from Canada and the United States suggest barriers to formal recognition persist, including the lack of emergency vehicle status, limited access to specialised training and insufficient preparation for managing traumatic events compared to other emergency personnel [21, 22]. Nonetheless, the frequency with which TTD are first on scene highlights the importance of revisiting what it is they do, what their needs are, their classification, thus ensuring that appropriate support and recognition can be improved.

## 4 Discussion

Findings from multiple studies are similar to other first responder's experience, such as firefighters, paramedics and police, where physical trauma is experienced in the form of violence from intoxicated consumers, being around death, and musculoskeletal pains and strains, all have an impact on PTSD [27, 28, 29]. Several interventions were identified to support firefighters, paramedics and police such as guided imagery and exercise, which helped allay anxiety and depression, and improve sleep, and well‐being [28]. In addition, these first responders also have access to workers compensation whilst recovering from physical trauma [29]. In view of the nature of physical trauma predisposing first responders to PTSD, in Australia it is recommended that resilience training be used to manage mental health challenges among firefighters, paramedics and police officers [30].

In contrast, no support, such as counselling or rehabilitation, was identified within the literature for TTD. This highlights the mismatch of available supports despite also attending road accidents and experiencing physical, verbal and emotional trauma through the work they do [2, 7]. As such, further research is required to explore the lived experience of TTD, with a particular emphasis on what supports are in place, where they seek help and what their identified physical needs are to ensure they remain safe at work. Given Dean et al. [1] described TTD as a ‘quartet of first responders’, this identified gap remains a palpable issue when compared to their first responder counterparts.

Psychological trauma was the second concern impacting TTD in the literature. These findings highlight witnessing fatal motor vehicle crash scenes as the main reason for psychological trauma and distress, and the preconceived trauma related to potentially rescuing friends or family is evident [4, 5]. Due to continuous exposure to such events, there is high risk of ongoing anxiety attending work and re‐traumatisation, which can be debilitating [4]. This can potentially promote TTD turnover and increase the statistics of mental health challenges. Therefore, understanding the lived experience can provide understanding and development of adaptive strategies, such as resilience to cope.

Similar to TTD, first responders are exposed to traumatic events which are deep‐rooted in their practice and detrimental to their mental well‐being [31]. According to literature, these include witnessing life‐threatening injuries, deaths of colleagues and being assaulted on the job [27, 32]. Continuous confrontation to these events may cause a challenge to psychological processing, resulting in distress, anxiety and the development of mental health conditions [31, 33]. As such, it is important for governments to consider a safety legislation that protects TTD and enhances their health and safety in the workplace.

Although the similarities in psychological trauma were identified for TTD and first responders, there is a vast difference in the availability of support services. For TTD, no literature identified specific available organisations to support their psychological challenges. However, studies conducted with firefighters, paramedics, the police suggest training for stress management, exercise programmes, skills training to increase the ability to cope with on‐the‐job stressors and mental health promotion [27, 33]. The risks undertaken to save people's lives and support people experiencing traumatic events are relatively similar among TTD and first responders. However, there is a palpable silence in research on this subject among TTD. The lack of evidence regarding psychological trauma for TTD compared to other first responders highlights the need to explore this issue further. This will enable greater understanding of their experience and what strategies, if any, are used to address psychological trauma when attending motor vehicle crashes. Similar strategies available for first responders could be trialled on TTD to identify effectiveness on their psychological challenges.

TTD experience physical and psychological trauma. Therefore, it is imperative to understand their coping strategies and the support services they can access to improve and maintain their well‐being. There is limited literature on how TTD cope with the challenges they encounter. Literature identified maladaptive coping strategies, such as alcohol and substance misuse, similar to first responders [4, 17]. The majority of studies reported that firefighters, paramedics and police confront their stressors, enabling identification of what adaptive coping strategies they can use, such as mindfulness, support from peers, family and friends, avoidance of the stressor and engaging in substance misuse to numb emotions [33, 34, 35]. Policies or frameworks could be developed with channels of support easily accessible for TTD, thus reducing the impact of trauma experienced.

Several studies identified that the tow truck industry has the highest fatality rate both physically and psychologically in the context of witnessing fatal car crashes or witnessing colleagues being injured or killed during work compared to other first responders, yet as a group, they are unrecognised by governments to assist in managing the consequences of performing this service to the community [4, 16, 22]. Given the similarities of roles during fatal motor vehicle accidents and physical and psychological experiences, it is crucial to implement accessible support services for TTD.

Safety training was one of the challenges experienced by TTD in the context of physical and mental well‐being. Numerous studies reported that the lack of safety training is one of the contributing factors for occupational hazards in the tow truck industry and that distressing crashes affect psychological safety in the tow truck industry [2, 22]. In comparison to firefighters, paramedics and police, Bevan et al. [36] postulated that regular exposure to critical incidents puts mental and physical well‐being at risk. Several studies identified strategies for safety, such as resilience training, access to employee assistance programmes, psychological first aid and a psychosocial safety climate, where managers are committed to reduce stress for the employees [36, 37, 38]. Although Madden [5] suggested the availability of workshops to obtain knowledge about mental health, there are limited opportunities compared to first responders. Given the similarities in the physical and psychological challenges, it is important to explore avenues of how safety measures can be implemented in the tow truck industry. This will ensure adequate well‐being for TTD and the ability to cope with their day‐to‐day challenges.

The conversation concerning safety and well‐being extends to a broader consideration of the role TTD plays in emergency response. The literature consistently highlights that TTD exhibit several traits commonly associated with first responders [1, 21]. However, their classification in this role is mitigated by factors, such as limited formal training and a lack of official recognition [21]. The debate regarding their status as first responders remains ongoing. Sanderson [22] observed that TTD are frequently dispatched alongside police and firefighters to assist with vehicle removal, indicating a coordinated response and shared responsibilities at crash scenes. Hence, it is imperative for the government to consider appropriate safety training to prepare TTD to attend traumatic motor crash scenes.

Despite not being formally trained in emergency procedures, TTD have been reported to perform lifesaving actions, such as administering CPR and assisting emergency services at crash scenes [4]. However, their lack of training in managing traumatic events and absence of legal authorisation to operate as emergency responders remain significant barriers to formal recognition [21, 22]. In response to these gaps, Chandler and Bunn [2] recommended legislative reform to formally include TTD within the category of first responders, alongside public awareness campaigns to promote their protection and recognition.

Given the physical and psychological risks TTD face, formal recognition as first responders or adjunct first responders warrants further examination. Such recognition may serve as a foundation for improving access to structured training, mental health support and workplace protections, resources that are routinely available to other emergency personnel. Addressing these gaps is essential not only for safeguarding the well‐being of TTD but also for ensuring that the critical role they play in emergency response is adequately supported and sustained.

### 4.1 Strengths and Limitations

A key strength of this review lies in its identification of a significant gap in the existing literature regarding TTDs’ role within the emergency response framework and the limited support services available to them for managing physical and psychological well‐being. By drawing attention to this under‐researched occupational group, the article contributes to a growing discourse on the need for broader recognition and targeted interventions. The synthesis of the available data highlights the parallels between TTD and first responders, reinforcing the argument for greater formal recognition and improved access to training, mental health resources and workplace protections.

However, one limitation of this study is the reliance on grey literature, which may introduce potential bias due to the non‐peer‐reviewed nature of some sources. This includes industry reports, media articles and organisational publications that, while informative, may lack the methodological rigour of academic research. The inclusion of grey literature was necessary given the scarcity of peer‐reviewed studies on this topic and provided valuable insights into lived experiences and operational realities often absent from formal research. Nonetheless, future studies would benefit from more robust empirical data and longitudinal research to validate and expand upon these findings.

## 5 Conclusion

Overall, the scoping review has highlighted that TTD are not formally recognised as first responders and lack the necessary training, legal authority to operate as emergency vehicles, and access to informal and formal support services, despite frequently being first to arrive at accident scenes compared to other members of the emergency ‘quartet’. Given the clear similarities in their roles, such as attending to vulnerable drivers and risking their lives to ensure the safety of others, it is reasonable to conclude that they experience comparable impacts on their physical and psychological well‐being. However, the support available to them remains inadequate when compared to emergency service counterparts. Within the limited research available, a significant gap has been identified that warrants further exploration to understand the physical and psychological experiences of TTD, how they manage these challenges and what supports are currently in place. Additionally, it is essential to examine what strategies could be implemented to enhance their well‐being and ensure a safer working environment within the tow truck industry. Recommendations include the development of targeted safety policies, legislative reform and emergency responder guidelines that formally recognise TTD as first responders, along with access to counselling services equivalent to those available to other emergency personnel.

## Author Contributions

**Brenda Ruvimbo Mutsvairo**: conceptualisation, methodology, formal analysis, writing – original draft. **Daniel Terry**: methodology, formal analysis, supervision, writing – review and editing. **Liz Ryan**: methodology, formal analysis, supervision, writing – review and editing. **Blake Peck**: supervision, writing – review and editing.

## Funding

The authors have nothing to report.

## Ethics Statement

The authors have nothing to report.

## Conflicts of Interest

The authors declare no conflicts of interest.

## Supporting information




**Table S1**: Search strings by database.
**Table S2**: Thematic analysis table.
**Table S3**: Overlapping themes.

## Data Availability

The data that support the findings of this study are available on request from the corresponding author. The data are not publicly available due to privacy or ethical restrictions.

## References

[1] L. Dean , W. Jame , and G. A. Ryan , “The Role of Towing Services at Motor Vehicle Crashes,” Medical Journal of Australia 2, no. 7 (1975): 252–255, 10.1136/bmj.313.7070.1448a. 1160789

[2] M. D. Chandler and T. L. Bunn , “Motor Vehicle Towing: An Analysis of Injuries in a High‐Risk Yet Understudied Industry,” Journal of Safety Research 71 (2019): 191–200, 10.1016/j.jsr.2019.10.006. 31862030

[3] FasterCapital , Rescue on Wheels: The AAA Tow Truck Lifesaver (FasterCapital 2023), https://fastercapital.com/content/Rescue‐on‐Wheels–The‐AAA‐Tow‐Truck‐Lifesaver.html.

[4] M. Calligeros , “The Forgotten Victims of Qld's Road Carnage,” Brisbane Times July 28, 2009, https://www.brisbanetimes.com.au/national/queensland/the‐forgotten‐victims‐of‐qlds‐road‐carnage‐20090727‐dyq0.html.

[5] T. Madden , How Do Drivers in the Industry Receive Emotional Support (Harvey's Towing, 2020), https://harveystowing.com.au/how‐do‐drivers‐in‐the‐industry‐receive‐emotional‐support/.

[6] C. Gray , Tow Truck Drivers: The Unsung Heroes (Drive, 2022), http://driveshops.com/tow‐truck‐drivers‐the‐unsung‐heroes/.

[7] L. Pitt , Tow Truck Drivers and Mental Stress (Lawyers.com, 2018), https://blogs.lawyers.com/attorney/workers‐compensation/tow‐truck‐drivers‐and‐mental‐stress‐50751/.

[8] H. Arksey and L. O'Malley , “Scoping Studies: Towards a Methodological Framework,” International Journal of Social Research Methodology 8, no. 1 (2005): 19–32, 10.1080/1364557032000119616.

[9] J. Popay , H. Roberts , A. Sowden , et al., Guidance on the Conduct of Narrative Synthesis in Systematic Reviews: A Product From the ESRC Methods Programme (Lancaster University, 2006), 10.13140/2.1.1018.4643.

[10] A. C. Tricco , E. Lillie , W. Zarin , et al., “PRISMA Extension for Scoping Reviews (PRISMA‐ScR): Checklist and Explanation,” Annals of Internal Medicine 169, no. 7 (2018): 467–473, 10.7326/M18-0850. 30178033

[11] M. J. Page , J. E. McKenzie , P. M. Bossuyt , et al., “The PRISMA 2020 Statement: An Updated Guideline for Reporting Systematic Reviews,” Bmj 372 (2021): n71, https://www.bmj.com/content/bmj/372/bmj.n71.full.pdf. 33782057 10.1136/bmj.n71 PMC8005924

[12] E. Delgado López‐Cózar , E. Orduna‐Malea , and A. Martín‐Martín , “ Google Scholar as a Data Source for Research Assessment ,” preprint, arXiv, June 18, 2018, 10.48550/arXiv.1806.04435.

[13] D. Levac , H. Colquhoun , and K. K. O'Brien , “Scoping Studies: Advancing the Methodology,” Implementation Science 5, no. 1 (2010): 69, 10.1186/1748-5908-5-69. 20854677 PMC2954944

[14] V. Braun and V. Clarke , Thematic Analysis: A Practical Guide (SAGE, 2021).

[15] A. Fugard and H. Potts , Thematic Analysis (SAGE Publications Ltd, 2019), https://methods.sagepub.com/foundations/thematic‐analysis.

[16] T. L. Bunn , S. Slavova , M. Chandler , N. Hanner , and M. Singleton , “Surveillance of Traffic Incident Management–Related Occupational Fatalities in Kentucky, 2005–2016,” Traffic Injury Prevention 19, no. 4 (2018): 446–453, 10.1080/15389588.2018.1432042. 29381397

[17] C. Yang , J. Liu , X. Li , and T. Barnett , “Analysis of First Responder‐Involved Traffic Incidents by Mining News Reports,” Accident Analysis & Prevention 192 (2023): 1–11, 10.1016/j.aap.2023.107261. 37572424

[18] Centres for Disease Control and Prevention , Two Man Crushed to Death While Hooking Up Disabled Propane Truck (Centres for Disease Control and Prevention, 2015), https://www.cdc.gov/niosh/face/stateface/ia/01ia002.html.

[19] Centres for Disease Control and Prevention , Tow Truck Operator Fatally Injured When Struck by a Box Truck While Assisting A Motorist‐Massachusetts (Centres for Disease Control and Prevention, 2017), https://www.cdc.gov/niosh/face/stateface/ma/15ma007.html.

[20] Chancey's Truck and Auto Salvage , Dangers of Tow Truck Driving (Chancey's Truck and Auto Salvage, 2022), https://www.chanceys.com/blog/dangers‐tow‐truck‐driving.

[21] R. C. Resch , Are Tow Trucks First‐Responders (American Towman Media, 2024), https://towindustryweek.com/index.php/12‐rates‐trade/6841‐are‐tow‐trucks‐first‐responders.

[22] B. Sanderson , “Tow Truck Driver Set to Discuss Psychological Stress, PTSD,” CBC November 23, 2015, https://www.cbc.ca/news/canada/nova‐scotia/tow‐truck‐drivers‐ptsd‐1.3328341.

[23] R. Bohn . Tow Truck Accidents (Golden State Lawyers, 2020), https://www.bohnlaw.com/2020/09/16/tow‐truck‐accidents/.

[24] B. C. Tefft , A. Wei , and R. Steinbach , Roadside Assistance Providers Fatally Struck by Vehicles at the Roadside: Incidence and Characteristics (AAA Foundation for Traffic Safety, 2024), https://aaafoundation.org/research/roadside‐assistance‐providers‐fatally‐struck‐by‐vehicles‐at‐the‐roadside‐incidence‐and‐characteristics/.

[25] SafeWork SA , “Companies Fined $950K Following Tow Truck Driver Death,” SafeWork SA September 15, 2022, https://safework.sa.gov.au/news‐and‐alerts/news/news/2022/companies‐fined‐$950k‐following‐tow‐truck‐driver‐death.

[26] Workplace Health and Safety Queensland , Worker Severely Injured by Failed Towing Equipment (Workplace Health and Safety Queensland, 2024), https://www.worksafe.qld.gov.au/news‐and‐events/alerts/incident‐alerts/2018/worker‐severely‐injured‐by‐failed‐towing‐equipment.

[27] N. F. Lewis‐Schroeder , K. Kieran , B. L. Murphy , J. D. Wolff , M. A. Robinson , and M. L. Kaufman , “Conceptualization, Assessment, and Treatment of Traumatic Stress in First Responders: A Review of Critical Issues,” Harvard Review of Psychiatry 26, no. 4 (2018): 216–227, 10.1097/hrp.0000000000000176. 29975339 PMC6624844

[28] J. Wild , S. El‐Salahi , and M. D. Esposti , “The Effectiveness of Interventions Aimed at Improving Well‐Being and Resilience to Stress in First Responders: A Systematic Review,” European Psychologist 25, no. 4 (2020): 252–271, 10.1027/1016-9040/a000402.

[29] S. E. Gray and A. Collie , “The Nature and Burden of Occupational Injury Among First Responder Occupations: A Retrospective Cohort Study in Australian Workers,” Injury 48, no. 11 (2017): 2470–2477, 10.1016/j.injury.2017.09.019. 28964511

[30] Parliament of Australia , Why First Responders? (Parliament of Australia, 2018), https://www.aph.gov.au/Parliamentary_Business/Committees/Senate/Education_and_Employment/Mentalhealth/Report/section?id=committees%2Freportsen%2F024252%2F26972#:~:text=The%20term%20`first%20responder'%20most,%2C%20often%20life%2Dthreatening%20situations.

[31] R. Jones , D. Jackson , and K. Usher , “First Responder Mental Health, Traumatic Events and Rural and Remote Experience,” Journal of Advanced Nursing 80, no. 2 (2024): 835–837, 10.1111/jan.15856. 37675883

[32] J. B. Casas and L. T. Benuto , “Breaking the Silence: A Qualitative Analysis of Trauma Narratives Submitted Online by First Responders,” Psychological Trauma: Theory, Research, Practice, and Policy 14, no. 2 (2022): 190–198, 10.1037/tra0001072. 34410810

[33] A. Greinacher , A. Nikendei , R. Kottke , et al., “Secondary Traumatisation in Psychosocial Emergency Care Personnel—A Longitudinal Study Accompanying German Trainees,” Health & Social Care in the Community 30, no. 3 (2022): 957–967, 10.1111/hsc.13258. 33370475

[34] E. Arble and B. B. Arnetz , “A Model of First‐Responder Coping: An Approach/Avoidance Bifurcation,” Stress and Health 33, no. 3 (2017): 223–232, 10.1002/smi.2692. 27500991 PMC6525630

[35] A. M. Díaz‐Tamayo , J. R. Escobar‐Morantes , and H. A. García‐Perdomo , “Coping Strategies for Exposure to Trauma Situations in First Responders: A Systematic Review,” Prehospital and Disaster Medicine 37, no. 6 (2022): 810–818, 10.1017/S1049023X22001479. 36326087

[36] M. P. Bevan , S. J. Priest , R. C. Plume , and E. E. Wilson , “Emergency First Responders and Professional Wellbeing: A Qualitative Systematic Review,” International Journal of Environmental Research and Public Health 19, no. 22 (2022): 14649, 10.3390/ijerph192214649. 36429361 PMC9691130

[37] K. Papazoglou , “Stress, Prevention, and Resilience Among First Responders,” International Journal of Environment Research and Public Health 20, no. 24 (2023): 7174, 10.3390/ijerph20247174. PMC10742391 38131724

[38] A. Hernandez Grande , F. Sharafizad , B. Farr‐Wharton , Y. Brunetto , and M. Richman , “Managing the Impact of Workplace Trauma for Australian First Responders: Harmonizing Policy and Practice,” Public Money & Management 45, no. 5 (2024): 414–423, 10.1080/09540962.2024.2401942.

