# Supplementary material for: The Experiences and Challenges Encountered by Tow Truck Driver's Attending Roadside Events: A PRISMA Scoping Review
Source: Public Health Chall. 2026 Jun 12;5(2):e70296. doi: 10.1002/puh2.70296 (PMC13261298; doi:10.1002/puh2.70296)
Supplement: Supplementary file 1 — Table S1: Search strings by database. Table S2: Thematic analysis table. Table S3: Overlapping themes. [file PUH2-5-e70296-s001.docx]

*Supplementary file 1*

Table S1: *Search strings by database*

| **Database** | **Search String** | **Number of articles** |
| --- | --- | --- |
| **PubMed** | ("tow truck" OR "towing truck operation" OR "towing vehicle" OR "crush towing" OR "crash towing") AND ("motor vehicle crash" OR "traumatic accident") OR (“accidents, traffic") AND ("mental health" OR "psychological support" OR "emotional wellbeing") | 430 |
| **Scopus** | TITLE-ABS-KEY ("tow truck" OR "towing truck operation" OR "towing vehicle" OR "crush towing" OR "crash towing") AND TITLE-ABS-KEY ("motor vehicle crash" OR "traumatic accident") OR (“accidents, traffic") AND TITLE-ABS-KEY ("mental health" OR "psychological support" OR "emotional wellbeing") | 267 |
| **CINAHL (via EBSCOhost)** | ("tow truck" OR "towing truck operation" OR "towing vehicle" OR "crush towing" OR "crash towing") AND ("motor vehicle crash" OR "traumatic accident") OR (“accidents, traffic") AND ("mental health" OR "psychological support" OR "emotional wellbeing") | 109 |
| **EBSCO – Newspaper Source Plus** | ("tow truck" OR "towing vehicle" OR "crash towing") AND ("accident" OR "collision") AND ("mental health" OR "psychological support" OR "emotional wellbeing") | 3814 |
| **Google Scholar** | "tow truck" OR "towing truck operation" OR "towing vehicle" OR "crush towing" OR "crash towing" "motor vehicle crash" OR "traumatic accident" OR “accidents, traffic" AND "mental health" OR "psychological support" OR "emotional wellbeing" | First 100 only |

*Supplementary file 2*

Table S2: *Thematic analysis table*

| **Author** | **Themes** |
| --- | --- |
| Bohn (2024) | Safety hazards:   - Fatigue related to long driving hours. - Drinking and driving. - Over speeding. - Lack of regular safety checks on the vehicle.   Traumatic incidents:   - Physical injuries related to accidents. |
| Bunn et al. (2018) | Occupational fatalities/Road hazard exposures:   - Out of the vehicle. - Performing the job. - Struck by another vehicle. |
| Calligeros (2009) | Witnessing traumatic events:   - No counselling provided/lack of support. - Seen more dead bodies than most Australian soldiers. - Giving up the job. - Attending to know individuals. - A sense of helplessness. - Substance and alcohol abuse, PTSD, and depression.   Seeking help:   - Not sought due to stigma, labelled as ‘hardened’ - Industry culture limits help-seeking. |
| CDC (2015) | Weather conditions:   - Snow covered roads.   Safety training:   - No safety program. - Crushed while hooking the vehicle. |
| CDC (2017) | Roadside hazards:   - Struck by a box truck. - Working on the roadside closest to the road. - Passing vehicle not slowing down when approaching a recovery vehicle.   Roadside structure:   - Narrow highway. - Narrow shoulder stopover.   Safety and training:   - Daily safety checklist. - On the job training. - A specific license was required. - Yellow warning lights. |
| Chance’s Truck and Auto Salvage (2022) | Roadside obstacles:   - Potholes, animals, and debris.   Weather and time:   - Storms and puddles. - Long hours of driving including nighttime. |
| Chandler and Bunn (2019) | Exposure to occupational hazards:   - Struck on the roadway as a pedestrian. - Struck while entering the vehicle. - Crushed beneath the customer’s vehicle.   Safety risks:   - Yellow lights but not indicating an emergency response. - Unexpected pinning beneath a vehicle. - Checking if the vehicle is properly supported during disengagement. - Lack of portable emergency devices.   Weather/Time/Climate hazards:   - Struck during low-light or dark hours. - Mostly cloudy conditions. |
| Dean et al. (1975) | Rapid response:   - Tow truck drivers arriving first on the crush scene. - No emergency vehicle status - Flashing hazard warning lights are required to minimize collisions and protect the accident scene. |
| Madden (2020) | Emotional support:   - Workplace mental and wellbeing awareness. - Reducing stigma. - Referrals to specialists, websites, and phone lines. |
| Pitt (2018) | Occupational hazards:   - Physical injuries related to hooking up the vehicles to the tow truck. - Being struck by other vehicles. - Physical assaults from customers.   Mental health support:   - Lack of formal emotional support after attending traumatic accident scenes. |
| Resch (2019) | First responders or not:   - Lack of a specialty siren. - Amber lights are not authorised as emergency. |
| Sanderson (2015) | Mental health support:   - Often overlooked. - Lack of government funds to provide psychological support. - lack of training to dela with traumatic scenes. - Available programs for private attendance. |
| Tefft et al. (2024) | Struck/Nearly struck:   - Occurred on a high-speed limit. - Outside the vehicle. - Standing or working outside the travel lanes or shoulder road.   Weather and time:   - Struck at nighttime. - Precipitation. - Wet or icy road.   Safety:   - Lack of required drivers’ license. - Driving with a suspended license. |
| Yang et al. (2023) | Severe injury by passing vehicle.   - Near miss - Line of duty death   Time/Location/Weather   - 8 am and 3-5 pm. - Snow, rain, and fog. |

*Supplementary File 3*

Table S3: *Overlapping themes*

| Author (year) | Exposure to trauma and high-risk working conditions | Psychological trauma and witnessing injuries and fatalities | Lack of professional recognition and support | Inadequate safety training and regulatory protections | Tow Truck Drivers as first responders |
| --- | --- | --- | --- | --- | --- |
| Bohn (1) | ✓ | ✓ |  |  |  |
| Bunn, Slavova (2) | ✓ | ✓ |  |  |  |
| Calligeros (3) | ✓ | ✓ | ✓ |  |  |
| Centres for Disease Control and Prevention (4) | ✓ | ✓ |  | ✓ |  |
| Centres for Disease Control and Prevention (5) | ✓ |  |  |  |  |
| Chancey’s Truck and Auto Salvage (6) | ✓ |  |  |  |  |
| Chandler and Bunn (7) | ✓ |  |  |  |  |
| Dean, Jame (8) |  |  |  |  | ✓ |
| Madden (9) |  | ✓ |  |  |  |
| Pitt (10) | ✓ | ✓ |  |  |  |
| Resch (11) |  |  |  |  | ✓ |
| Sanderson (12) | ✓ | ✓ | ✓ |  |  |
| Tefft (13) | ✓ |  |  |  |  |
| Yang, Liu (14) | ✓ |  |  |  |  |
